# LncRNA MALAT1 Promotes Tumor Angiogenesis by Regulating MicroRNA-150-5p/VEGFA Signaling in Osteosarcoma: *In-Vitro* and *In-Vivo* Analyses

**DOI:** 10.3389/fonc.2021.742789

**Published:** 2021-10-07

**Authors:** Selvaraj Vimalraj, Raghunandhakumar Subramanian, Anuradha Dhanasekaran

**Affiliations:** ^1^ Centre for Biotechnology, Anna University, Chennai, India; ^2^ Department of Pharmacology, Saveetha Dental College and Hospital, Saveetha Institute of Medical and Technical Sciences (SIMATS), Chennai, India

**Keywords:** lncRNA MALAT1, microRNA-150-5p, VEGFA, osteosarcoma, angiogenesis

## Abstract

The present study aims to analyze the expression of long noncoding RNA (lncRNA) metastasis-associated lung adenocarcinoma transcript 1 (MALAT1) in human osteosarcoma (OS) cells and to investigate its role in OS-induced angiogenesis. MALAT1 expression in OS cells was significantly higher than in normal osteoblasts. The functional analysis indicated that MALAT1 appears to enhance OS-induced angiogenesis, *in vitro* and *in vivo* analyses, endothelial cell proliferation and migration, chick embryo angiogenesis assay, and zebrafish xenograft model. Mechanistically, silencing MALAT1 downregulated vascular endothelial growth factor A (VEGFA) expression and upregulated miR-150-5p expression in OS cells, and MALAT1-mediated angiogenic induction by VEGFA in OS microenvironment. Moreover, MALAT1 directly targeted miR-150-5p and miR-150-5p directly target VEGFA in OS. Overexpression of miR-150-5p downregulates VEGFA expression in OS. More notably, we showed that MALAT1 induced angiogenesis in OS microenvironment by upregulating the expression of VEGFA *via* targeting miR-150-5p. Overall, our findings suggest that MALAT1 promotes angiogenesis by regulating the miR-150-5p/VEGFA signaling in OS microenvironment. The findings of the molecular mechanisms of MALAT1 in tumor angiogenesis offer a new viewpoint on OS treatment.

## Introduction

In children and adolescents, osteosarcoma (OS) is the most prevalent primary malignant tumor formed in the bone. Patients with osteosarcoma are usually treated with surgery and extensive adjuvant chemotherapy. However, patients with OS who are treated with surgery alone have a survival rate of 15%–17%. In the last 30 years, survival rates for OS patients with metastasis or relapse have remained largely stable (1, 2). Angiogenesis is a critical component in the formation and progression of OS, according to growing data (3). Several regulators, including noncoding RNAs such as long noncoding RNAs (LncRNAs) and microRNAs, are involved in the OS-induced angiogenesis. LncRNAs are a type of noncoding RNA with a length of more than 200 nucleotides. Increasing evidence suggests that lncRNAs play a significant role in cancer, resulting in abnormal gene expression and contributing to the growth of a variety of human tumors. The role of LncRNAs in tumor-induced angiogenesis has also been recognized (4). For instance, colorectal cancer-associated lncRNA (CCAL), a long noncoding RNA, increases tumor angiogenesis in OS through regulating the ANGPTL4/miR-29b signaling (5). Through the miR-138/VEGF/HIF-1 signaling, lncRNA H19 stimulates glioma angiogenesis (6).

In many human malignancies, the lncRNA MALAT1 serves as an oncogenic gene. MALAT1 is recently reported to stimulate OS progression and metastasis. For example, through activating miR-140-5p/HDAC4 signaling, the lncRNA MALAT1 regulates OS cell proliferation and apoptosis (7). LncRNA MALAT1 promotes OS lung metastasis by sponging miR-202 (8). In OS cells, a MALAT1/miR-26a-5p/FOXO1 feedback loop regulates migration and proliferation (9). However, the biological relevance and molecular mechanisms of MALAT1 in OS-induced angiogenesis remain unknown. The expression of lncRNA MALAT1 in OS cells was measured in this work, and the function and regulatory mechanisms of lncRNA MALAT1 in human OS-induced angiogenesis was examined. Finally, our findings revealed that the lncRNA MALAT1 increases angiogenesis in OS microenvironment through modulating the miR-150-5p/VEGFA axis.

## Materials and Methods

### Cell Culture and Transfection

Osteosarcoma cell lines (SaOS2, MG63, and HOS) were obtained from NCCS, India. Prof. Suvro Chatterjee, Anna University, Chennai, India, kindly offered the human endothelial cell line, EA.hy926 cells. Cells were cultured in a standard condition of DMEM containing 10% FBS at 37°C and 5% CO_2_. For transient transfection, MG63/SAOS2 cells (5 × 10^4^) were plated on 18 mm coverslips and cultured to reach ~70% confluence. Transient transfection of miRNAs and siRNAs was carried out as previously described (10). Thermo Fisher Scientific (Waltham, MA, USA) provided lncRNA MALAT1-specific silencer siRNA (Si-MALAT1) and nonsilencer siRNA (scrambled siRNA/Si-control): si-MALAT1, 5′-GCAGAGGCATTTCATCCTT-3′ and si-control, 5′-TTCTCCGAACGTGTCACGT-3′, The transfection reagent (X-treme Gene Transfection Reagent, Roche, Basel, Switzerland) was combined with Si-MALAT1 (20 nM) or Si-control (20 nM), and the transfection was carried out as directed by the manufacturer’s instructions. Similarly, miR-150-5p mimic (50 nM) or control miRNA (50 nM) (Applied Biosystems, Waltham, MA, USA) were mixed with transfection reagent and transfection was carried out. The cells were transfected and then cultured up to 24 h to reach 100% confluence.

### Endothelial Cell Proliferation and Migration

EA.hy926 cells were plated at a density of 3 × 10^4^ cm^2^ in 24-well plates, and cell proliferation was measured using the MTT assay (11). Briefly, after 24 h of miRNA/SiRNA transfection, a conditioned medium was obtained from SaOS2/MG63 cells. The prepared conditioned media were used to culture EA.hy926 cells, and endothelial cell proliferation and morphology were analyzed. After treatment, the fluorescein diacetate (FDA) solution, 30 g/ml was exposed with EA.hy926 cells and morphology was analyzed under fluorescent microscope. For the wound scratch assay, EA.hy926 cells (1 × 10^6^ cells/ml) were cultured in a 24-well culture plate for 24 h to reach confluence. To create a wound, the monolayer was scratched with a 20-µl tip, and the cells were treated with conditioned media. Bright field images were captured under an inverted microscope after treatment. Using ImageJ software, the rate of wound closure was determined. Also, under MG63 and SaOS2 cell-conditioned media treatment, Boyden chamber assay was utilized to assess EA.hy926 cell migration, as described previously (11). The upper well was loaded with EA.hy926 cells, while the lower well with conditioned medium obtained from MG63/SaOS2 cells after siRNA transfection. The membrane was fixed and stained with DAPI after the experiment, and the migratory cells were analyzed under a fluorescence microscope. The proliferation of the loaded cells in the Boyden chamber was calculated by counting EA.hy926 cells before and after the experiment.

### Real-Time RT-PCR

Total RNA was obtained using the Trizol reagent of Invitrogen (Waltham, MA, USA) according to the manufacturer’s instructions. According to the manufacturer’s instructions, cDNA was produced using a Reverse Transcriptase kit (Invitrogen). A SYBR premix Ex Taq II (TIi RNase H plus) was used for real-time PCR analysis (Takara, Kusatsu, Japan). For the cell line study, GAPDH and U6 were used to normalize mRNA and miRNA expressions, respectively. For CAM assay-based mRNA expression, the β-actin was employed. **Supplementary Table 1** lists the primer sequences utilized in this research. Reverse transcription was performed using a micro Script II RT kit (Qiagen, Hilden, Germany) for mature miRNA. The 50-µl reaction volume was made up of 1× miScript Universal Primer, 1× miScript primer assay, 1× QuantiTect SYBR Green PCR Master Mix, template cDNA, and RNase free water for expression. ΔΔCt method of relative quantification was used to calculate relative miRNA/mRNA expression (12).

### Human VEGFA Quantification by ELISA Kit

The cells (SaOS2 and MG63) were transfected with miRNA/siRNA and grown for up to 24 h. According to the manufacturer’s instructions, the conditioned medium was prepared and VEGFA quantified using a human VEGFA ELISA Kit (Elabscience, Wuhan, China).

### Western Blot

Following the methodology published elsewhere, Western blot analysis was performed (12). Total cell lysates were extracted with RIPA buffer including a protease inhibitor cocktail (Sigma-Aldrich, St. Louis, MO, USA), and 20 μg of protein was utilized for Western blot analysis with VEGFA (1:1,000) and α-tubulin (1:1,000) antibodies (Santa Cruz Biotechnology, Dallas, TX, USA). Similarly, total lysates from Zebrafish embryos were extracted, and Western blot analysis was done using the antibodies Tie-2 (1:1,000) (R&D Systems, Minneapolis, MN, USA), VEGFA (1:1,000) (Abcam, Cambridge, UK), α-SMA (1:1,000) (GeneTex, Irvine, CA, USA), and α -tubulin (1:1,000) (R&D Systems). Supersignal West Dura Extended Duration Substrate (Thermo Scientific) was employed to detect the protein bands.

### Luciferase Reporter Assay

The luciferase reporter assay was carried out according to the manufacturer’s protocol using the pmirGLO dual-luciferase miRNA target expression vector (Promega, Madison, WI, USA). The wild and mutant VEGFAs/MALAT1s sense and antisense primers with an internal *Not*I site were annealed and cloned between *Pme*I and *Xba*I restriction sites in vector. The *Not*I restriction enzyme was used to identify the clones that contained the VEGFA/MALAT1 3′-UTR sequence. SaOS2/MG63 cells were transfected with wild/mutant VEGFA/MALAT1 3′-UTR reporter plasmid (200 ng) and miRNA (50 nM) using Lipofectamine 2000 transfection reagent (Invitrogen). A dual-luciferase reporter assay kit was used to assess luciferase activity after 24 h. The ratio of Renilla/firefly luciferase activity was measured (10).

### Egg Yolk Angiogenesis Assay

The Government Poultry Station in Chennai provided preincubated white Leghorn chick (*Gallus gallus*) eggs. The eggs were incubated under standard conditions of 37°C and 60% humidity. Hamburger Hamilton (HH) stage 22 embryos were used in all of the investigations. The eggs were cracked open and placed in foam containers with the embryos face up, with a permeable polyethylene lining covering them. The shell-less embryos were kept at 35.5°C with a humidity of 68% (13). On the vascular bed, 18 mm coverslips containing MG63 cells with or without transfection were placed, allowing the cells to come into direct touch with the vascular bed. The images were taken using a stereomicroscope connected with a Magnus digital camera and analyzed using picasa3 and AngioQuant software after treatment for up to 6 h (14).

### Animal Model and MG63 Cell Transplantation

Zebrafish breeding and maintenance were undertaken in an in-house facility, according to recognized methods (10). Institutional animal ethical committee of Saveetha University in Chennai, India, approved all protocols (BRULAC/SDCH/SIMATA/IAEC.3-2021/067). MG63 cells were initially transfected with si-MALAT1 or si-control, as reported in the *Materials and Methods* section. The cells were labeled with CellTracker™ Green 5-chloromethylfluorescein diacetate (CMFDA) (Thermo Fisher) as per the manufacturer’s protocol after 24 h of transfection and then implanted in embryos as stated (10, 15). MG63 cells (~1,000 cells) were injected into 2 days postfertilization embryos. The yolk sac near the Cuvier duct was injected with a glass needle supplied with ~10 nl of cell suspension solution by a Femtojet injector (Eppendorf). The implanted embryos were allowed to settle for 1 h at 28°C before being kept at 35°C. Fluorescence microscopy was used to image the MG63 cells 24 h after implantation.

### Statistical Analysis

The means ± SD are used to represent all of the data. To compare quantitative variables, a one-way analysis of variance or a Student’s *t*-test was utilized. To determine the correlation between the groups, a Pearson correlation analysis was used. It was considered that *p* < 0.05 was statistically significant.

## Results and Discussion

### LncRNA MALAT1 Expression in OS Suppress Proliferation and Migration of Endothelial Cells

**Figure 1:**
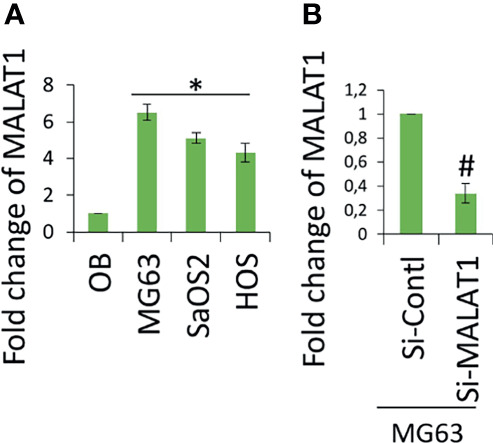
lncRNA MALAT1 is overexpressed in OS cell lines. **(A)** Real-time RT-PCR was used to detect the expression level of lncRNA MALAT1 in different OS cells lines, MG63, SaOS2, and HOS, and the expression was compared with normal osteoblast differentiated from hDPSCs. Asterisk indicates significant increase compared with osteoblast. **(B)** Si-MALAT1 decrease MALAT1 expression in MG63 cells. Si-MALAT1/Si-control was transfected into MG63 cells, and after 24 h, the real-time RT-PCR analysis was used to detect MALAT1 expression. Number sign indicates significant decrease compared with si-control.

**Figure 2:**
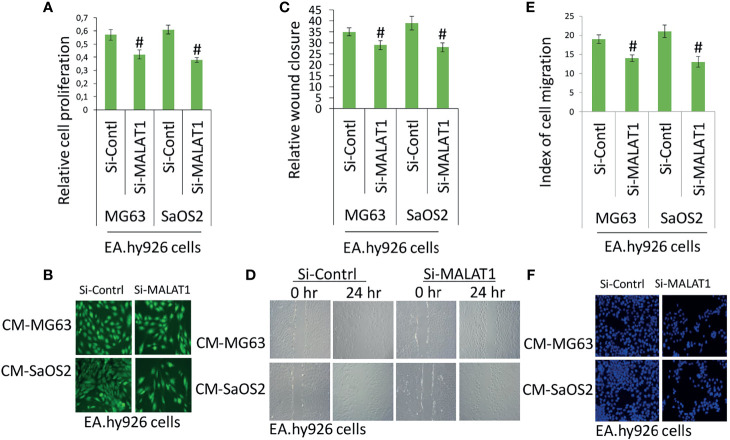
Knockdown of MALAT1 in OS decreased endothelial cell proliferation and migration. After transfection of si-MALAT1 or si-control in MG63 and SaOS2 cells, the conditioned medium was prepared. EA.hy926 cells were exposed with the prepared conditioned medium and cell proliferation and migration was analyzed. **(A)** EA.hy926 cell proliferation was analyzed my MTT assay, and **(B)** cell morphology was analyzed by FDA staining after 24 h of treatment. **(C)** Similarly, scratch wound assay was performed and analyzed relative to wound closure and **(D)** representative images of wound healing assay. **(E)** Index of cell migration was analyzed by Boyden chamber assay and **(F)** shows representative image of DAPI staining of migrated cells. Number sign indicates significant decrease compared with si-control.

Accumulating evidence indicates that MALAT1 appears to serve as an oncogene in a variety of human malignancies, including OS, and angiogenesis is a critical process in the formation and progression of osteosarcoma. However, not much research on MALAT1 regulation in OS-induced tumor angiogenesis has been done. Therefore, real-time RT-PCR analysis was used to detect MALAT1 expression in OS cell lines in order to assess its level. The MALAT1 expression level was elevated in the OS cell line than in the osteoblasts differentiated from human dental pulp stem cells (hDPSCs), as shown in **Figure 1A**. MG63 and SaOS2 cells were transfected with si-MALAT1 or si-control, and MALAT1 expression was measured to determine the involvement of MALAT1 in OS-induced angiogenesis. The results of real-time RT-PCR demonstrated that Si-MALAT1 was effective in knocking down MALAT1 in MG63 and SaOS2 cells (**Figure 1B**). To further investigate the role of MALAT1 in angiogenesis in OS, the cell proliferation, morphology, and migration assays were performed in EA.hy926 cells treated with conditioned medium from SaOS2 and MG63 cells transfected with si-MALAT1 or si-control. Cell proliferation was analyzed by MTT assay (**Figure 2A**), morphology assessed by FDA staining (**Figure 2B**), and migration was analyzed by scratch wound (**Figures 2C, D**) and Boyden chamber assay (**Figures 2E, F**). The results showed that decreased endothelial cell proliferation and migration were observed in the si-MALAT1 group compared with si-control group (**Figures 2A–F**).

### LncRNA MALAT1 Positively Regulates VEGFA and Negatively Regulates miR-150-5p Expression in OS

**Figure 3:**
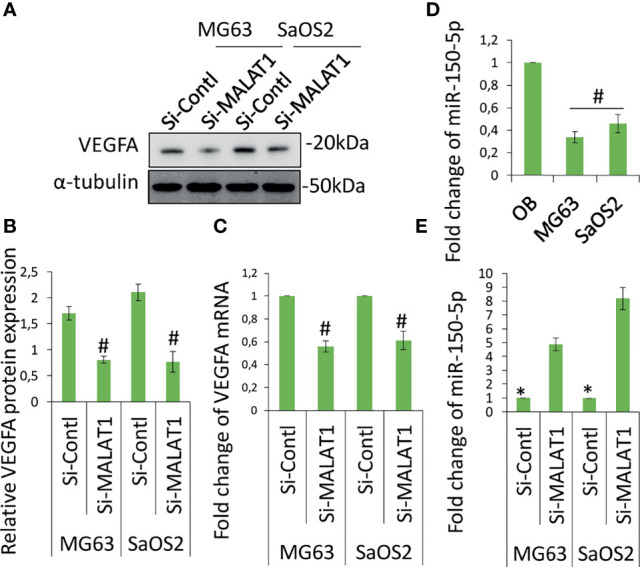
Knockdown of MALAT1 in OS decreased VEGFA and increased miR-150-5p expression. VGFA and miR-150-5p expression was analyzed in MG63 and SaOS2 cells after transfection of si-MALAT1 or si-control. After 24 h, VEGFA protein was analyzed by Western blot analysis **(A)**. **(B)** The medium was collected and secreted VEGFA level was analyzed by ELISA kit. Similarly, **(C)** VEGFA mRNA expression was analyzed by real-time RT-PCR. **(D)** miR-150-5p expression was analyzed by real-time RT-PCR in OS cell lines and compared with normal osteoblast differentiated from hDPSCs. **(E)** After transfection of si-MALAT1 and si-control in MG63 and SaOS2 cells, the expression of miR-150-5p was analyzed by real-time RT-PCR. Asterisk indicates significant increase compared with si-control, and Number sign indicates significant decrease compared with si-control.

The angiogenesis-related gene, VEGFA level was analyzed in MG63 and SaOS2 cells and cell culture supernatants of MG63 and SaOS2 cells *via* Western blot (**Figure 3A**) and ELISA (**Figure 3B**). **Figures 3A, B** shows that knocking down MALAT1 by si-MALAT1 transfection greatly reduced VEGFA expression and secretion level in MG63 and SaOS2 cells but had no impact on HGF or VEGFD/C (data not shown). Furthermore, si-MALAT1-reduced VEGFA mRNA expression considerably (**Figure 3C**). VEGFA is a well-known regulator of tumor angiogenesis. VEGFA is found to be released by various tumors including OS to control angiogenesis by stimulating the endothelial cells in its microenvironment. Tumor activates angiogenesis by secreting VEGFA (11, 16). Collectively, MALAT1 promotes angiogenesis in OS by regulating VEGFA. Previous research has shown that lncRNAs play a significant role in cancer growth and progression by acting as miRNA decoys that control gene expression. The probable interactions between lncRNAs and miRNAs were predicted to further examine the molecular mechanisms involved in MALAT1-induced VEGFA expression. MALAT1 promotes OS cell metastasis and proliferation (17). MALAT1 targets miR-150-5p in osteoarthritis thereby regulating cartilage cell apoptosis, extracellular matrix degradation, and osteoarthritis development by the activation of miR-150-5p/AKT3 signaling (18). Similarly, MALAT1-miR-150-5p interactions and regulatory mechanisms were reported in a variety of conditions, including systemic juvenile idiopathic arthritis (19), airway smooth muscle cell proliferation (20), endoplasmic reticulum stress regulation (21), and pregnancy-induced hypertension (22). In colorectal cancer, miR-150-5p inhibits cell growth and metastasis by directly targeting VEGFA (23, 24). LncRNA TNK2-AS1 regulated oxidized low-density lipoprotein-stimulated human aortic smooth muscle cell proliferation and migration by VEGFA/miR-150-5p axis (25). miR-150-5p/VEGFA signaling mediates extravillous trophoblast cell migration and angiogenesis (26). Hence, we speculate that MALAT1 may play a role in the control of miR-150-5p, instructing OS to release VEGFA in their microenvironment in order to induce angiogenesis. The initial miR-150-5p analysis in OS cells, it was found to be considerably downregulated in MG63 and SaOS2 cells relative to normal osteoblasts differentiated from hDPSCs (**Figure 3D**). MALAT1 knockdown dramatically inhibited the expression of miR-150-5p in MG63 and SaOS2 cells, as shown in **Figure 3E**. MALAT1 upregulates VEGFA and downregulates miR-150-5p expression in OS, according to the findings.

### miR-150-5p Directly Targets VEGFA and LncRNA MALAT1 Directly Targets miR-150-5p in OS

**Figure 4:**
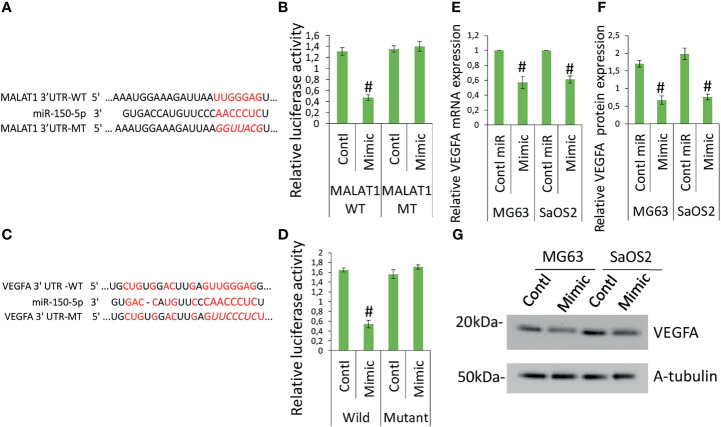
MALAT1 targets miR-150-5p and miR-150-5p targets VEGFA in OS. MALAT1 directly targets miR-150-5p in OS. **(A)** The interactions between MALAT1 and miR-150-5p were predicted. **(B)** Dual-luciferase reporter assays were performed in MG63 and SaOS2 cells after cotransfection MALAT1 3′-UTR wild/mutant with miR-150-5p mimic or control miR. Number sign indicates significant decrease compared with other form of cotransfection. Similarly, **(C)** the interactions between miR-150-5p and VEGFA were predicted. **(D)** Dual-luciferase reporter assays were performed in MG63 and SaOS2 cells after cotransfection VEGFA 3′-UTR wild/mutant with miR-150-5p mimic or control miR. Number sign indicates significant decrease compared with other form of cotransfection. miR-150-5p effects on VEGFA expression in OS was analyzed by transfecting miR-150-5p mimic or control miR in MG63 and SaOS2 cells. miR-150-5p mimic suppressed VEGFA mRNA **(E)**, VEGFA-secreted protein level **(F)**, and VEGFA protein expression **(G)** in MG63 and SaOS2 cells. Number sign indicates significant decrease compared with control miR.

The target sequence for miR-150-5p on the 3′-UTR of MALAT1 is shown elsewhere (19–21). **Figure 4A** shows the predicted binding sites. Next, reporter vectors containing miR-150-5p binding regions in the wild MALAT1 3-UTR and mutant MALAT1 3-UTR were used to measure relative luciferase activity. In MG63 cells cotransfected with miR-150-5p mimic and wild MALAT1 3′-UTR, the relative luciferase activity was reduced, as shown in **Figure 4B**. The result showed that there is a direct interaction between MALAT1 and miR-150-5p in OS. MALAT1 interaction with miR-150-5p is documented in several studies. MALAT1 involvement in OS promotion is well reported previously (27, 28). The regulation and interaction between MALAT1 and miR-150-5p have been confirmed using various molecular techniques like RNA pull down, ribonucleoprotein immunoprecipitation analysis, and luciferase reporter assay and in various studies (18–22).

To further investigate the function of miR-150-5p in the expression of VEGFA induced by MALAT1, the possible interactions between miR-150-5p and VEGFA were predicted. The expected results suggested that VEGFA contained a miR-150-5p-binding site (**Figure 4C**). Next, reporter vectors containing miR-150-5p-binding regions in the wild VEGFA 3-UTR and mutant VEGFA 3-UTR were used to measure relative luciferase activity. In MG63 cells cotransfected with miR-150-5p mimic and wild VEGFA 3′-UTR, the relative luciferase activity was reduced, as shown in **Figure 4D**. Furthermore, in MG63 cells, miR-150-5p mimic dramatically reduced VEGFA protein (**Figure 4E**) and mRNA (**Figure 4F**), as well as secretory levels of VEGFA (**Figure 4G**). The findings revealed that in OS, miR-150-5p and VEGFA had a negative interaction. Taken together, our findings imply that VEGFA is a direct miR-150-5p target gene. The interaction between miR-150-5p and VEGFA is documented in various studies. MALAT1 involvement in OS promotion is well-reported previously (27, 28). MALAT1 regulation of miR-150-5p is also reported in various conditions (18–22). miR-150-5p regulation of VEGFA is also reported in various conditions (23–26). Hence, we investigated whether MALAT1 regulation of miR-150-5p/VEGFA signaling plays a role in OS-induced angiogenesis.

### LncRNA MALAT1 Regulates VEGFA-Mediated Tumor Angiogenesis in OS by Targeting miR-150-5p

**Figure 5:**
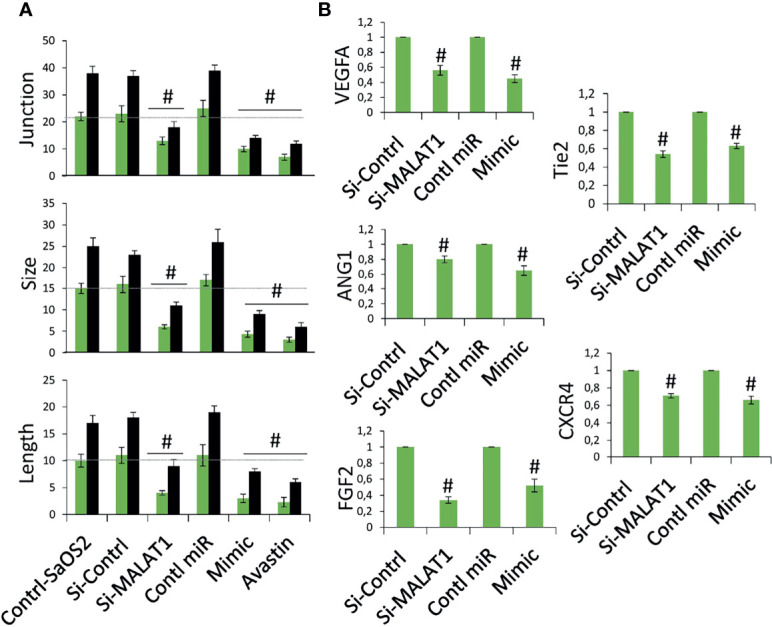
Knockdown of MALAT1 and overexpression of miR-150-5p in OS suppress tumor angiogenesis by regulating VEGFA. **(A)** MG63 cells were transfected with si-MALAT/si-control/miR-150-5p mimic/control miR. After 24 h, the cells were exposed on CAM and the development of blood vessels was examined. In si-MALAT1 and miR-150-5p mimic transfection, angiogenic characteristics such as blood vessel size, junction, and length were reduced. Avastin, a VEGF inhibitor, was also employed as a positive control. Number sign indicates significant increase compared with control. For statistical analysis, only 6 h of treatment were considered. **(B)** Real-time RT-PCR was used to examine the angiogenic markers. A similar procedure was used, and the vascular bed from CAM was dissected and subjected to FGF2, VEGFA, CXCR4, Tie2, and ANG1 mRNA expression. Number sign indicates significant increase compared with control.

The next step was to see if MALAT1 overexpression in OS can control tumor angiogenesis *via* miR-150-5p/VEGFA signaling. A chick embryo CAM model was employed to analyze this. Using chick embryos, we recently established a novel model to test the angiogenic regulation of tumor (13). MG63 cells were grown in coverslips and transfected with si-MALAT/si-control, miR-150-5p mimic, or control miRNA for 24 h, then inversely kept on CAM bed at day 4. In a recent investigation, we discovered that a dose of MG63 of 50,000 is ideal for a possible tumor angiogenic investigation (13). The CAM vascular bed image was obtained after 6 h of treatment and analyzed for angiogenic characteristics using picasa3 and AngioQuant software. The results were compared between 2 and 6 h. The results in comparison with si-control-transfected cells, si-MALAT1-transfected MG63 cells revealed lower blood vessel junction, length, and size (**Figure 5A**). Similarly, miR-150-5p mimic transfected MG63 cell exposure also reduced these angiogenic parameters compared with control miRNA-transfected cells. Additionally, inhibition of VEGFA by Avastin in MG63 cells showed decreased tumor angiogenic effect. In addition, total RNA was extracted from the CAM vasculature after 6 h of treatment and subjected to real-time RT-PCR analysis for the expression of angiogenic markers VEGFA, ANG1, Tie2, CXCR4, and FGF2 (**Figure 5B**). VEGFA, angiopoietin 1 (ANG1), Tie2, CXC motif chemokine receptor 4 (CXCR4), and fibroblast growth factor (FGF2) are well-known angiogenic marker genes (11, 29). As expected, the angiogenic markers, VEGFA, ANG1, Tie2, CXCR4, and FGF2 mRNA expression were decreased in si-MALAT1-transfected MG63 cell treatment group compared with si-control-transfected MG63 cell treatment group. Similarly, these markers were reduced in miR-150-5p mimic-transfected MG63 cell treatment group compared with the control miRNA-transfected MG63 cell treatment group.

**Figure 6:**
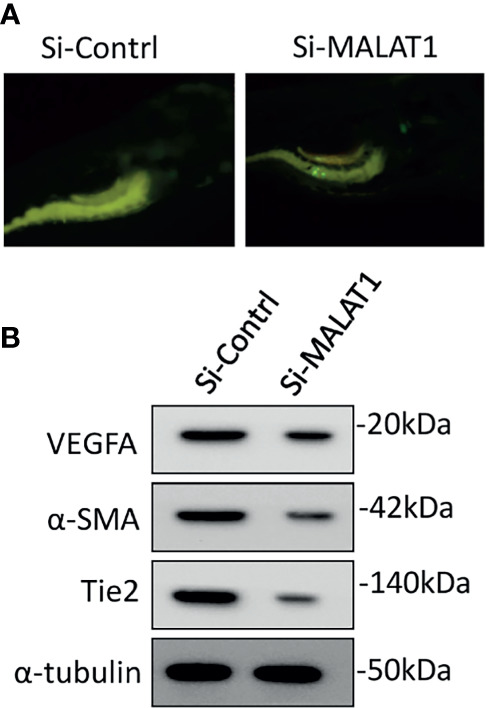
Knockdown of MALAT1 suppressed OS-induced angiogenesis in zebrafish xenograft model. **(A)** CellTracker™ Green CMFDA-labeled MG63 cells were injected into 2 days of postfertilization, which were screened after 24 h **(B)** After 24 h of cell injection, a whole lysate was collected from the embryo and subjected to Western blot analysis using the antibodies indicated. As an internal loading control, α-tubulin was used.

The involvement of MALAT1 in OS-induced angiogenesis in zebrafish was then investigated. The zebrafish tumor xenograft model is based on the embryos being implanted with human tumor cells (10, 15). Initially, MG63 cells were transfected with si-MALAT1 or si-control, then tagged with CellTracker™ Green CMFDA and implanted into 2 days postfertilization zebrafish embryos. With the use of a tracking dye, the transplanted MG63 cells were easily tracked. Efficient homing of MG63 cells around the gastrointestinal area is found after 24 h of injection, confirming tumor existence (**Figure 6A**). The embryo survival rate following implantation was measured for up to 6 days, and tumor cell injection resulted in roughly 60% survival compared with 1× PBS injection. In MG63 cells, however, there was no difference in survival ratio between si-MALAT1 and si-control transfection. The angiogenic marker expressions was examined by Western blot after 24 h of MG63 cell xenografting in zebrafish embryos (**Figure 6B**). VEGFA, α-SMA, and Tie2 are all known to have a role in tumor angiogenesis and are employed as angiogenic markers (29, 30). The expression of VEGFA, α-SMA, and Tie2 proteins was lower in embryos implanted with si-MALAT1-transfected MG63 cells than in embryos implanted with si-control-transfected MG63 cells. As a result of the *in vivo* findings, MALAT1 signaling in OS may modulate tumor angiogenesis. Overall, MALAT1 promotes VEGFA expression in OS cells to induce angiogenesis by targeting miR-150-5p. Furthermore, earlier studies convincingly demonstrated the relationship between MALAT1 and miR-150-5p, as well as its role in tumor and tumor-induced angiogenesis (31–34). Similarly, the interaction of miR-150-5p and VEGFA, as well as its role in tumor and tumor-induced angiogenesis is also studied (23–26). In addition, the expression of MALAT1, miR-150-5p, and VEGFA in various tumors regulates angiogenesis in the microenvironment (23–26, 31–34). Therefore, analyzing and targeting MALAT1/miR-150-5p/VEGFA signaling, as well as other biomolecules, may be a better therapeutic strategy for preventing angiogenesis in OS microenvironment.

## Conclusions

**Figure 7:**
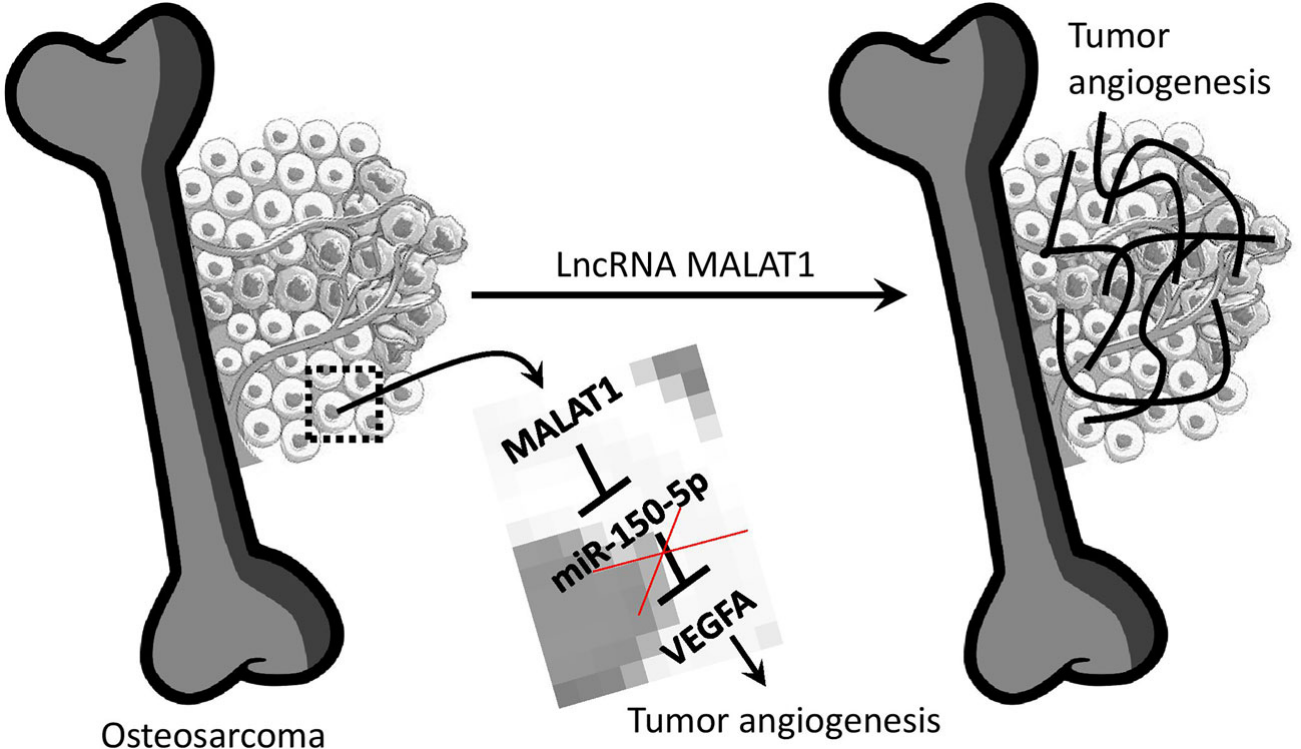
Overview of lncRNA MALAT1 regulation of OS-induced angiogenesis. MALAT1 expression upregulates VEGFA by targeting miR-150-5p. This regulation saves VEGFA from miR-150-5p regulation.

To summarize, MALAT1 is overexpressed in OS, and silencing MALAT1 in the OS suppresses endothelial cell proliferation and migration. Additionally, in the absence of MALAT1, VEGFA expression was reduced while miR-150-5p expression was increased in OS. As expected, the direct target study revealed that MALAT1 and miR-150-5p, as well as miR-150-5p and VEGFA, have a negative interaction. Furthermore, overexpression of miR-150-5p was found to reduce VEGFA expression in OS. Moreover, in the CAM and zebrafish xenograft models, silencing MALAT1 and overexpression of miR-150-5p reduced OS-induced angiogenesis. Overall, this is the first work to show that MALAT1 is an oncogenic lncRNA that increases angiogenesis in the OS microenvironment by modulating the miR-150-5p/VEGFA axis (**Figure 7**). As a result of this discovery, MALAT1 could be considered a potential therapeutic target for OS patients.

## Data Availability Statement

The raw data supporting the conclusions of this article will be made available by the authors, without undue reservation.

## Ethics Statement

The animal study was reviewed and approved by BRULAC/SDCH/SIMATS/IAEC/3-2021/067.

## Author Contributions

SV and RS performed all the experiments. SV drafted the manuscript. AD provided technical support for the work. SV secured the funding, designed the work, analyzed data, and approved the final submitted manuscript. All authors contributed to the article and approved the submitted version.

## Funding

This work was also supported by the Department of Science and Technology, Inspire Faculty Program, Government of India (Grant No: DST/INSPIRE/04/2017/002913 to SV).

## Conflict of Interest

The authors declare that the research was conducted in the absence of any commercial or financial relationships that could be construed as a potential conflict of interest.

## Publisher’s Note

All claims expressed in this article are solely those of the authors and do not necessarily represent those of their affiliated organizations, or those of the publisher, the editors and the reviewers. Any product that may be evaluated in this article, or claim that may be made by its manufacturer, is not guaranteed or endorsed by the publisher.

## References

[1] Kansara M Teng MW Smyth MJ Thomas DM. Translational Biology of Osteosarcoma. Nat Rev Cancer (2014) 14(11):722–35. doi: 10.1038/nrc3838 25319867

[2] Meyers PA Healey JH Chou AJ Wexler LH Merola PR Morris CD. Addition of Pamidronate to Chemotherapy for the Treatment of Osteosarcoma. Cancer (2011) 117(8):1736–44. doi: 10.1002/cncr.25744 PMC3059356 21472721

[3] Vimalraj S Bhuvaneswari S Lakshmikirupa S Jyothsna G Chatterjee S. Nitric Oxide Signaling Regulates Tumor-Induced Intussusceptive-Like Angiogenesis. Microvasc Res (2018) 119:47–59. doi: 10.1016/j.mvr.2018.04.001 29649432

[4] Sheng SR Wu JS Tang YL Liang XH. Long Noncoding RNAs: Emerging Regulators of Tumor Angiogenesis. Future Oncol (2017) 13(17):1551–62. doi: 10.2217/fon-2017-0149 28513194

[5] Chen S Yang M Chang S. LncRNA CCAL Promotes Angiogenesis Through Regulating the MiR-29b/ANGPTL4 Axis in Osteosarcoma. Cancer Manag Res (2020) 12:10521–30. doi: 10.2147/CMAR.S272230 PMC7591080 33122950

[6] Liu ZZ Tian YF Wu H Ouyang SY Kuang WL. LncRNA H19 Promotes Glioma Angiogenesis Through miR-138/HIF-1α/VEGF Axis. Neoplasma (2020) 67(1):111–8. doi: 10.4149/neo_2019_190121N61 31777264

[7] Sun Y Qin B. Long Noncoding RNA MALAT1 Regulates HDAC4-Mediated Proliferation and Apoptosis *via* Decoying of miR-140-5p in Osteosarcoma Cells. Cancer Med (2018) 7(9):4584–97. doi: 10.1002/cam4.1677 PMC6144160 30094957

[8] Zhang J Piao CD Ding J Li ZW. LncRNA MALAT1 Facilitates Lung Metastasis of Osteosarcomas Through miR-202 Sponging. Sci Rep (2020) 10(1):12757. doi: 10.1038/s41598-020-69574-y 32728178 PMC7391763

[9] Wang J Sun G. FOXO1-MALAT1-miR-26a-5p Feedback Loop Mediates Proliferation and Migration in Osteosarcoma Cells. Oncol Res (2017) 25(9):1517–27. doi: 10.3727/096504017X14859934460780 PMC7841132 28160461

[10] Vimalraj S Subramanian R Saravanan S Arumugam B Anuradha D. MicroRNA-432-5p Regulates Sprouting and Intussusceptive Angiogenesis in Osteosarcoma Microenvironment by Targeting PDGFB. Lab Invest (2021) 101(8):1011–25. doi: 10.1038/s41374-021-00589-3 33846539

[11] Vimalraj S Saravanan S Raghunandhakumar S Anuradha D. Melatonin Regulates Tumor Angiogenesis *via* miR-424-5p/VEGFA Signaling Pathway in Osteosarcoma. Life Sci (2020) 256:118011. doi: 10.1016/j.lfs.2020.118011 32592723

[12] Vimalraj S Partridge NC Selvamurugan N. A Positive Role of microRNA-15b on Regulation of Osteoblast Differentiation. J Cell Physiol (2014) 229(9):1236–44. doi: 10.1002/jcp.24557 PMC4038448 24435757

[13] Vimalraj S Pichu S Pankajam T Dharanibalan K Djonov V Chatterjee S. Nitric Oxide Regulates Intussusceptive-Like Angiogenesis in Wound Repair in Chicken Embryo and Transgenic Zebrafish Models. Nitric Oxide (2019) 82:48–58. doi: 10.1016/j.niox.2018.11.001 30439561

[14] Niemistö A Dunmire V Yli-Harja O Zhang W Shmulevich I. Robust Quantification of *In Vitro* Angiogenesis Through Image Analysis. IEEE Trans Med Imaging (2005) 24:549–53. doi: 10.1109/TMI.2004.837339 15822812

[15] Chiavacci E Rizzo M Pitto L Patella F Evangelista M Mariani L. The Zebrafish/Tumor Xenograft Angiogenesis Assay as a Tool for Screening Anti-Angiogenic miRNAs. Cytotechnology (2015) 67:969–75. doi: 10.1007/s10616-014-9735-y PMC4628928 24947063

[16] Xie L Ji T Guo W. Anti-Angiogenesis Target Therapy for Advanced Osteosarcoma (Review). Oncol Rep (2017) 38(2):625–36. doi: 10.3892/or.2017.5735 PMC5562076 28656259

[17] Wang Y Zhang Y Yang T Zhao W Wang N Li P. Long Non-Coding RNA MALAT1 for Promoting Metastasis and Proliferation by Acting as a ceRNA of miR-144-3p in Osteosarcoma Cells. Oncotarget (2017) 8(35):59417–34. doi: 10.18632/oncotarget.19727 PMC5601743 28938647

[18] Zhang Y Wang F Chen G He R Yang L. LncRNA MALAT1 Promotes Osteoarthritis by Modulating miR-150-5p/AKT3 Axis. Cell Biosci (2019) 9:54. doi: 10.1186/s13578-019-0302-2 31304004 PMC6600894

[19] Jiang H Zhu M Wang H Liu H. Suppression of lncRNA MALAT1 Reduces Pro-Inflammatory Cytokines Production by Regulating miR-150-5p/ZBTB4 Axis Through JAK/STAT Signal Pathway in Systemic Juvenile Idiopathic Arthritis. Cytokine (2021) 138:155397. doi: 10.1016/j.cyto.2020.155397 33341002

[20] Lin L Li Q Hao W Zhang Y Zhao L Han W. Upregulation of LncRNA Malat1 Induced Proliferation and Migration of Airway Smooth Muscle Cells *via* miR-150-Eif4e/Akt Signaling. Front Physiol (2019) 10:1337. doi: 10.3389/fphys.2019.01337 31695627 PMC6817469

[21] Liu L Yan LN Sui Z. MicroRNA-150 Affects Endoplasmic Reticulum Stress *via* MALAT1-miR-150 Axis-Mediated NF-κb Pathway in LPS-Challenged HUVECs and Septic Mice. Life Sci (2021) 265:118744. doi: 10.1016/j.lfs.2020.118744 33181172

[22] Ou M Zhao H Ji G Zhao X Zhang Q. Long Noncoding RNA MALAT1 Contributes to Pregnancy-Induced Hypertension Development by Enhancing Oxidative Stress and Inflammation Through the Regulation of the miR-150-5p/ET-1 Axis. FASEB J (2020) 34(5):6070–85. doi: 10.1096/fj.201902280R 32246794

[23] Chen X Xu X Pan B Zeng K Xu M Liu X. miR-150-5p Suppresses Tumor Progression by Targeting VEGFA in Colorectal Cancer. Aging (Albany NY) (2018) 10(11):3421–37. doi: 10.18632/aging.101656 PMC6286841 30476901

[24] Cai T Cui X Zhang K Zhang A Liu B Mu JJ. LncRNA TNK2-AS1 Regulated Ox-LDL-Stimulated HASMC Proliferation and Migration *via* Modulating VEGFA and FGF1 Expression by Sponging miR-150-5p. J Cell Mol Med (2019) 23(11):7289–98. doi: 10.1111/jcmm.14575 PMC6815783 31468685

[25] Chen X Zeng K Xu M Hu X Liu X Xu T. SP1-Induced lncRNA-ZFAS1 Contributes to Colorectal Cancer Progression *via* the miR-150-5p/VEGFA Axis. Cell Death Dis (2018) 9(10):982. doi: 10.1038/s41419-018-0962-6 30250022 PMC6155123

[26] Zeng Y Wei L Lali MS Chen Y Yu J Feng L. miR-150-5p Mediates Extravillous Trophoblast Cell Migration and Angiogenesis Functions by Regulating VEGF and MMP9. Placenta (2020) 93:94–100. doi: 10.1016/j.placenta.2020.02.019 32250744

[27] Wang S Ren L Shen G Liu M Luo J. The Knockdown of MALAT1 Inhibits the Proliferation, Invasion and Migration of Hemangioma Endothelial Cells by Regulating MiR-206 / VEGFA Axis. Mol Cell Probes (2020) 51:101540. doi: 10.1016/j.mcp.2020.101540 32084582

[28] Li Z Dou P Liu T He S. Application of Long Noncoding RNAs in Osteosarcoma: Biomarkers and Therapeutic Targets. Cell Physiol Biochem (2017) 42(4):1407–19. doi: 10.1159/000479205 28715796

[29] Ucuzian AA Gassman AA East AT Greisler HP. Molecular Mediators of Angiogenesis. J Burn Care Res (2010) 31(1):158–75. doi: 10.1097/BCR.0b013e3181c7ed82 PMC2818794 20061852

[30] Saravanan S Vimalraj S Pavani K Nikarika R Sumantran VN. Intussusceptive Angiogenesis as a Key Therapeutic Target for Cancer Therapy. Life Sci (2020) 252:117670. doi: 10.1016/j.lfs.2020.117670 32298741

[31] Yadav MK Manoli NM Vimalraj S Madhunapantula SV. Unmethylated Promoter DNA Correlates With p53 Expression and Apoptotic Levels Only in Vitamin B9 and B12 Deficient Megaloblastic Anemia But Not in Non-Megaloblastic Anemia Controls. Int J Biol Macromol (2018) 109:76–84. doi: 10.1016/j.ijbiomac.2017.12.070 29246873

[32] Yao MY Zhang WH Ma WT Liu QH Xing LH Zhao GF. Long non-Coding RNA MALAT1 Exacerbates Acute Respiratory Distress Syndrome by Upregulating ICAM-1 Expression *via* microRNA-150-5p Downregulation. Aging (Albany NY) (2020) 12(8):6570–85. doi: 10.18632/aging.102953 PMC7202495 32315984

[33] Ren K Ni Y Li X Wang C Chang Q Li Y. Expression Profiling of Long Noncoding RNAs Associated With Vasculogenic Mimicry in Osteosarcoma. J Cell Biochem (2019) 120(8):12473–88. doi: 10.1002/jcb.28514 30825232

[34] Zhang ZC Tang C Dong Y Zhang J Yuan T Tao SC. Targeting the Long Noncoding RNA MALAT1 Blocks the Pro-Angiogenic Effects of Osteosarcoma and Suppresses Tumour Growth. Int J Biol Sci (2017) 13(11):1398–408. doi: 10.7150/ijbs.22249 PMC5715523 29209144

