# Supplementary material for: LncRNA MALAT1 Promotes Tumor Angiogenesis by Regulating MicroRNA-150-5p/VEGFA Signaling in Osteosarcoma: In-Vitro and In-Vivo Analyses
Source: Front Oncol. 2021 Oct 7;11:742789. doi: 10.3389/fonc.2021.742789 (PMC8529043; doi:10.3389/fonc.2021.742789)
Supplement: Supplementary file 1 [file Table_1.docx]

Supplementary table 1. List of primer sequence used for the study

| Gene |  | 5’ to 3’ |
| --- | --- | --- |
| Chicken FGF-2 | Forward | AGAGGAGTAGTATCAATCAAAG |
|  | Reverse | TGCCACATACCAATCAGAGT |
| Chicken ANG-1 | Forward | GAGTCTGGTCACTCGGCAAA |
|  | Reverse | CTAGGCTGCCATCTTCTCGG |
| Chicken ANG-2 | Forward | GAGCAGACCCGCAAATTAAC |
|  | Reverse | GAACTGTCCAACCTCCTCCA |
| Chicken VEGFA | Forward | TGAGGGCCTAGAATGTGTCC |
|  | Reverse | TCTTTTGACCCTTCCCCTTT |
| Chicken  β-actin | Forward | TCTGACTGACCGCGTTACTC |
|  | Reverse | CCATCACACCCTGATGTCTG |
| Human VEGFA | Forward | CAAGTGGTCCCAGGCTGCAC |
|  | Reverse | AGCTCATCTCTCCTATGTGC |
| Human GAPDH | Forward | TTGATGTCATCATACTTGGCAGGT |
|  | Reverse | CAG TCAAGGCTGAGAATGGGA |
| MALAT1 | Forward | ATCTGCAAAACAAAAACCCCT |
|  | Reverse | GTCTCCGAAGACACAGAGACCT |
| U6 | Forward | CTCGCTTCGGCAGCACA |
|  | Reverse | AACGCTTCACGAATTTGCGT |
